# Predictors and a Prediction Model for Central Cervical Lymph Node Metastasis in Papillary Thyroid Carcinoma (cN0)

**DOI:** 10.3389/fendo.2021.789310

**Published:** 2022-01-27

**Authors:** Xin Gao, Wenpei Luo, Lingyun He, Juan Cheng, Lu Yang

**Affiliations:** ^1^Department of Breast and Thyroid Surgery, Second Affiliated Hospital of Chongqing Medical University, Chongqing, China; ^2^Department of Ultrasound, Second Affiliated Hospital of Chongqing Medical University and Chongqing Key Laboratory of Ultrasound Molecular Imaging, Chongqing, China; ^3^Scientific Research and Education Section, Chongqing Health Center for Women and Children, Chongqing, China

**Keywords:** papillary thyroid carcinoma, cervical lymph node metastasis, cervical lymph node dissection, predictor, prediction model

## Abstract

**Objectives:**

To screen out the predictors of central cervical lymph node metastasis (CLNM) for papillary thyroid carcinoma (PTC) and establish a prediction model to guide the operation of PTC patients (cN0).

**Methods:**

Data from 296 PTC patients (cN0) who underwent thyroid operation at the Second Affiliated Hospital of Chongqing Medical University were collected and retrospectively analyzed. They were divided into two groups in accordance with central CLNM or not. Their information, including ultrasound (US) features, BRAF^V600E^ status, and other characteristics of the two groups, was analyzed and compared using univariate and multivariate logistic regression analyses, and the independent predictors were selected to construct a nomogram. The calibration plot, C-index, and decision curve analysis were used to assess the prediction model’s calibration, discrimination, and clinical usefulness.

**Results:**

A total of 37.8% (112/296) of PTC patients had central CLNM, and 62.2% (184/296) did not. The two groups were compared using a univariate logistic regression analysis, and there were no significant differences between the two groups in sex, aspect ratio, boundary, morphology, hypoechoic nodule, thyroid peroxidase antibody, or tumor location (P>0.05), and there were significant differences between age, tumor size, capsule contact, microcalcifications, blood flow signal, thyroglobulin antibodies (TgAb), and BRAF gene status (P<0.05). A multivariate logistic regression analysis was performed to further clarify the correlation of these indices. However, only tumor size (OR=2.814, 95% Cl=1.634~4.848, P<0.001), microcalcifications (OR=2.839, 95% Cl=1,684~4.787, P<0.001) and TgAb (OR=1.964, 95% Cl=1.039~3,711, P=0.038) were independent predictors of central CLNM and were incorporated and used to construct the prediction nomogram. The model had good discrimination with a C-index of 0.715. An ROC curve analysis was performed to evaluate the accuracy of this model. The decision curve analysis showed that the model was clinically useful when intervention was decided in the threshold range of 16% to 80%.

**Conclusion:**

In conclusion, three independent predictors of central CLNM, including tumor size (> 1.0 cm), US features (microcalcifications), and TgAb (positive), were screened out. A visualized nomogram model was established based on the three predictors in this study, which could be used as a basis of central cervical lymph node dissection (CLND) for PTC patients (cN0).

## Introduction

Papillary thyroid carcinoma (PTC) is the most common endocrine carcinoma. Over the past 30 years, the morbidity of thyroid carcinoma has doubled or more in many countries, including China (1). Most of the increased incidence was PTC, especially PTC with a maximum tumor diameter (MTD) < 1 cm (2). The central cervical lymph nodes of PTCs with diameters less than 1 cm are often clinically negative (cN0). Practically, the incidence of central cervical lymph node metastasis (CLNM) in PTCs is still quite high. Studies have shown that the incidence can reach 20% - 80% (3, 4). Due to the excellent survival rate of PTC, the scope of surgical resection of PTC is controversial.

At present, no examination can precisely indicate central CLNM. A study showed that the sensitivity, specificity, positive predictive value, negative predictive value, and accuracy of preoperative ultrasound (US) diagnosis for PTC patients with central cervical lymph node metastasis were 35.3%, 88.6%, 83.2%, 47.4%, and 56.6%, respectively (5), which are not sufficiently reliable for diagnosing central CLNM. Due to the lack of accurate preoperative diagnosis of central CLNM and the relatively high incidence of metastasis, routine prophylactic central lymph node dissection (RCLND) is often performed in patients with clinically negative central lymph nodes (cN0) in China. The benefits of RCLND for PTC patients (cN0) are manifold. First, RCLND may facilitate the diagnosis of accurate TNM staging, the use of I^131^ therapy, and thyroid-stimulating hormone (TSH) suppressive therapy (6, 7). Second, RCLND may reduce recurrence during postoperative follow-up (8). In addition, RCLND helps to avoid reoperation and any related complications (7, 9). Nonetheless, there is still controversy as to whether RCLND should be used in PTC patients (cN0), especially in T1 and T2 stage carcinomas (10). The incidence of lymph node metastasis was 40.1%, including central, lateral, and mediastinal metastasis incidences of 56.6%, 34.1%, and 9.3%, respectively (11). Accordingly, nearly half of the PTC patients have no central CLNM, and they did not benefit from RCLND. Moreover, the incidence of permanent hypoparathyroidism and permanent laryngeal nerve injury after total thyroidectomy ranged from 1 to 2% and 0 to 5.5%, respectively. It increased to 0 to 14.3% and 0 to 5.7% separately when the patients (cN0) underwent RCLND (12). Therefore, routine RCLND poses additional surgical risks, and it is crucial to screen PTC patients (cN0) for predictors of central CLNM and establish relevant prediction models to achieve accurate central cervical lymph node dissection (CLND).

Many studies have focused on the risk factors for central CLNM in patients (cN0). However, the results were inconsistent. Age is important for PTC staging. Traditionally, patients over 45 years of age have a slightly worse prognosis and an increased recurrence rate (13, 14). Some studies have also shown that age is a risk factor for central CLNM. In contrast, other studies have reported a higher incidence of central CLNM in patients younger than 45 years of age (15, 16). Tumor size is also a key factor in TNM staging, with larger tumors more likely to be aggressive. It has been reported that the larger the tumor is, the higher the incidence of metastasis; however, there is no consensus on the threshold of size. Sex is also an important indicator; females have a higher incidence of PTC. However, some studies have shown that male sex is a risk factor for central CLNM because males are more likely to engage in unhealthy behaviors, such as smoking and alcohol consumption (17, 18). US features, including spotty microcalcifications and aspect ratio, are vital information for differentiating malignant nodules from benign nodules. Meanwhile, they are considered risk factors for central CLNM (19, 20). Although controversial, RCLND is still recommended in China. The purpose of our study was to filter out important predictors from more medical information and to develop a prediction model (nomogram) for accurate central CLND. Nomograms establish prediction models based primarily on biological and clinical variables, which have been used to predict tumor-related events, including death and other high risks (21). The prediction results can help clinicians to improve patient prognosis by making appropriate clinical interventions for possible events (22).

## Material and Methods

### Patients

All patients were examined and treated at the Second Affiliated Hospital of Chongqing Medical University (Chongqing, China). The inclusion criteria were as follows: (1) patients who underwent primary thyroid surgery and central CLND; (2) patients with histopathologically confirmed PTC; and (3) patients who underwent preoperative thyroid function tests without history of thyroxine treatment [including thyroid-stimulating hormone (TSH, reference: 0.35–5.00 μIU/ml, negative: <=5.00 μIU/ml, and high: TSH >5.00), thyroglobulin (Tg, reference: 1.40–78.00 μg/L, low: <1.4 μg/L, normal: 1.40–78.00 μg/L, and high: > 78 μg/L), thyroglobulin antibody (TgAb, reference: 0.00–115.00 IU/ml, negative: 0.00–115.00 IU/ml, and positive: >115.00 IU/ml), and thyroid peroxidase antibody (TPOAb, reference: 0.00–34.00 IU/ml, negative: 0.00–34.00 IU/ml, and positive: > 34.00 IU/ml)], US, and BRAF^V600E^ mutation tests in addition to history taking. The exclusion criteria were as follows: (1) patients with other pathological types of thyroid cancer, such as medullary carcinoma and follicular carcinoma; (2) patients with other concomitant malignancies, such as breast cancer and nasopharyngeal carcinoma; (3) patients with multiple nodules where at least two of them were diagnosed as PTC; and (4) patients with incomplete medical data. Based on the inclusion and exclusion criteria, all 296 patients included in this study were diagnosed with PTC by fine-needle aspiration biopsy (FNAB) and BRAF^V600E^ mutation analysis prior to surgery between January 2019 and July 2020.

### Ultrasonography

Prior to FNAB, nodules were examined with color Doppler US (Philips IU22, L12-5 line array probe, 5-12 MHz, Philips Medical), showing US features such as nodule size (>1.0 cm/<1.0 cm, maximal diameter), boundary (clear/unclear), shape (regular/irregular), internal echo, aspect ratio (≤1/>1), capsular contact (yes/no), microcalcifications (yes/no) and blood flow (poor/rich).

### US-Guide FNAB

**Figure 1:**
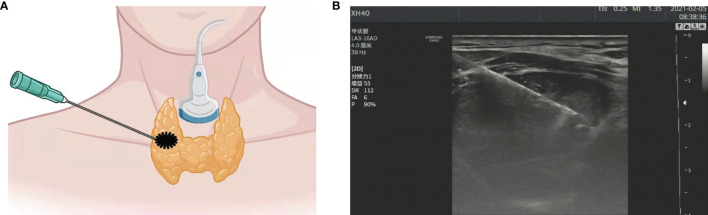
**(A)** Schematic diagram of ultrasound (US)-guided fine needle aspiration biopsy (thyroid nodule). **(B)** US image of fine needle aspiration (thyroid nodule).

While the patients were in a supine position with their backs raised and their heads tilted back, US was used to determine the insertion site, angle, and depth. The probe was fixed, with the puncture point on one side of the probe, and the puncture route was set at 30–40° to the long axis of the probe surface (**Figure 1**). Once the fine needle was positioned inside the thyroid nodule, the needle was repeatedly inserted and rotated 10 to 20 times and then quickly withdrawn. The specimen was fixed onto a slide and visually designated suitable for further analysis and microscopic observations before delivery to the Department of Pathology of the Second Affiliated Hospital of Chongqing Medical University (Chongqing, China) for cytological examination and subsequently forwarded to the Molecular Diagnostic Laboratory of the Second Affiliated Hospital of Chongqing Medical University for BRAF^V600E^ mutation analysis.

### BRAF^V600E^ Mutation Analysis

The reaction mixture was thawed at room temperature, mixed on a vortex for 15 sec, and subsequently centrifuged at 2,000 × g for 15 sec at room temperature. The reaction mixture (35 µl) was mixed with 0.4 µl Taq enzyme and later dispensed into a PCR tube (ice bath). Next, 5 µl DNA sample (2-5 ng), 5 µl positive control, and 5 µl negative control were added separately to each PCR tube. The PCR tubes were centrifuged at 2,000 × g for 1 min at room temperature, and a thermocycler (CFX96; Bio–Rad Laboratories, Inc.) was used. The following thermocycling conditions were used for quantitative PCR: initial denaturation at 95°C for 5 min; 15 cycles at 95°C for 25 sec, 64°C for 20 sec and 72°C for 20 sec; and an additional 31 cycles at 93°C for 25 sec, 60°C for 35 sec and 72°C for 20 sec. Carboxyfluorescein (FAM) and hexachlorofluorescein (HEX) signals were detected at 60°C during the final set of cycling conditions. The 2^−ΔΔCq^ method was used to quantify the relative amount of DNA (23). If the Cq value of the FAM signal of the sample was ≥28, the sample was considered negative for the BRAF^V600E^ mutation; if the Cq value of the FAM signal of the sample was <28, the sample was deemed to be positive for the BRAF^V600E^ mutation according to the manufacturer’s protocol (24).

### Thyroid Surgery and Pathologic Analysis

During the operations, the nodules were sent for frozen sections (5 µm thick sections, under optical microscopy at x400 magnification) and confirmed as PTC. After rediagnosis, these patients underwent thyroid resection (ipsilateral lobectomy) and ipsilateral CLND (including pretracheal, ipsilateral paratracheal, and prelaryngeal lymph nodes), and the central lymph nodes were also sent for frozen sections to confirm central CLNM. If there was central CLNM, total thyroidectomy was performed. After the operations, the remaining specimens (nodules and central cervical lymph nodes) were sent for paraffin sections (hematoxylin and eosin, 5-µm-thick sections, under optical microscopy at 400x magnification) and finally confirmed as PTC and central CLNM.

### Statistical Analysis

The statistical analysis was performed using SPSS 26.0. A univariate logistic regression analysis was performed to determine the predictors of central CLNM, and the variables with statistical significance on the univariate analysis were analyzed by a multivariate logistic regression analysis (a two-sided P value <0.05) to indicate a significant difference. The prediction nomogram based on the multivariate logistic regression analysis results was developed using R software (Version 4.1.0; https://www.r-project.org/). A calibration curve was plotted to assess the calibration of the nomogram. Harrell’s C-index (area under the curve, AUC) was measured to quantify the discrimination of the nomogram through the training cohort and further confirmed through two internal validations. The training cohort was divided into two internal validation cohorts (100 patients included in Validation 1 and 95 patients included in Validation 2) by random sampling. A decision curve analysis was performed to determine the clinical usefulness of the nomogram by quantifying the net benefit at the different thresholds.

## Results

### Patients’ Characteristics

**Figure 2:**
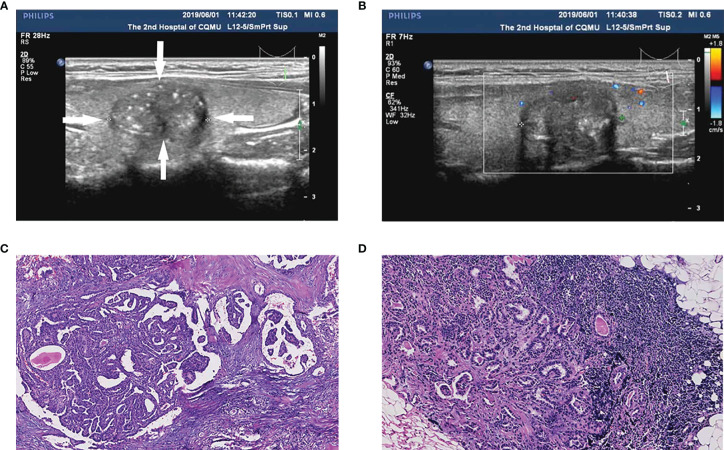
A papillary thyroid carcinoma (PTC) patient (35-year-old male, 1.5 cm nodule) with central cervical lymph node metastasis (CLNM) and positive BRAF^V600E^ mutation. **(A)** The thyroid nodule had a low echo, microcalcifications, and irregular morphology on US images. **(B)** Color doppler flow image (CDFI) showed a lack of blood supply inside the nodule. **(C)** Pathologic examination confirmed the diagnosis of PTC. **(D)** Pathologic examination confirmed the diagnosis of central CLNM.

**Figure 3:**
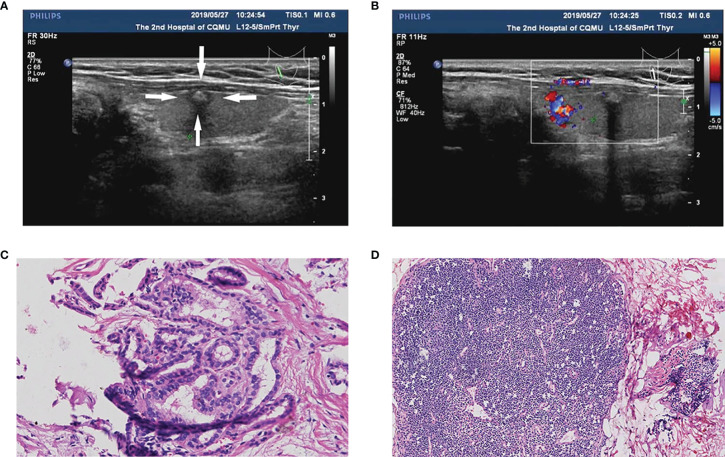
A PTC patient (55-year-old female, 0.6 cm nodule) with non-central CLNM and positive BRAF^V600E^ mutation. **(A)** The thyroid nodule had low-echo, microcalcifications, capsule contact, and unclear boundary on US image. **(B)** CDFI showed a lack of blood supply inside the nodule. **(C)** Pathologic examination confirmed the diagnosis of PTC. **(D)** Pathologic examination confirmed the diagnosis of non-central CLNM.

After pathological examination, a total of 296 patients were finally confirmed to have PTC, among which 75 (25.3%) were men, and 221 (74.7%) were women. Of the PTCs, 119 (40.2%) were in the left lobe, 162 (54.7%) were in the right lobe, and 9 (5.1%) were in the isthmus (**Table 1**). Central CLNM was confirmed by postoperative pathology. Of all patients, 112 (37.8%) had central CLNM (**Figure 2**), while 184 (62.2%) did not (**Figure 3**).

**Table 1:** Univariate analysis of the relationship between medical information and central CLNM.

Medical information	Lymph node metastasis		χ^2^	P
	Yes (n =112)	No (n = 184)		
Age (year)			5.731	0.022
≤40	69 (44.23)	87 (55.87)		
>40	43 (30.71)	97 (69.29)		
Gender			3.329	0.074
female	77 (34.84)	144 (65.16)		
male	35 (46.67)	40 (53.33)		
Tumor size (cm)			25.027	<0.001
≤1.0	57 (28.22)	145 (71.78)		
>1.0	55 (58.51)	39 (41.49)		
Aspect ratio			2.069	0.179
<1	76(35.35)	139 (64.65)		
≥1	36 (44.44)	45 (55.56)		
Tumor location			0.142	0.931
left	45 (37.82)	74 (62.18)		
right	62 (38.27)	100 (61.73)		
isthmic	5 (33.33)	10 (66.67)		
Capsule contact			13.295	<0.001
present	100 (43.29)	131 (56.71)		
absent	12 (18.46)	53 (81.54)		
Microcalcifications			21.279	<0.001
absent	36 (24.66)	110 (75.34)		
present	76 (50.67)	74 (49.33)		
Boundary			1.226	0.306
unclear	80 (40.00)	120 (60.00)		
clear	32 (33.33)	64 (66.67)		
Morphology			2.223	0.195
Irregular	106 (39.11)	165 (60.89)		
regular	6 (24.00)	19 (76.00)		
Low echo			0.985	0.322
no	20 (44.44)	25 (55.56)		
yes	92 (36.65)	159 (63.35)		
BRAF^V600E^			4.839	0.038
Mutation	101 (37.55)	168 (62.45)		
Wild	11 (40.74)	16 (59.26)		
Blood flow signal			4.782	0.033
poor	31 (29.52)	74 (70.48)		
rich	81 (42.41)	110 (57.59)		
TSH			1.356	0.342
negative	106 (37.20)	179 (62.80)		
high	6 (54.54)	5 (45.46)		
Tg			0.555	0.758
low	15 (39.47)	23 (60.53)		
normal	88 (36.97)	150 (63.03)		
high	9 (45.00)	11 (55.00)		
TgAb			5.103	0.033
positive	29 (50.88)	28 (49.12)		
negative	83 (34.73)	156 (65.27)		
TPOAb			2.899	0.100
positive	28 (47.46)	31 (52.54)		
negative	84 (35.44)	153 (64.56)		

Thyroid function tests feature: TSH (reference: 0.35–5.00 μIU/ml), TgAb (reference: 0.00–115.00 IU/ml), TPOAb (reference: 0.00–34.00 IU/ml), Tg (reference: 1.40–78.00 μg/L).

### Univariate Logistic Regression Analysis

The relationships between medical information and central CLNM are presented in **Table 1**. There were significant differences in age (≤40 years old, P<0.05), tumor size (>1.0 cm, P<0.05), capsule contact (P<0.05), microcalcifications (present, P<0.05), blood flow signal (poor, P<0.05), TgAb (positive, P<0.05) and BRAF^V600E^ status (mutation, P<0.05) between the CLNM group and the non-CLNM group. Meanwhile, sex, aspect ratio, boundary, morphology, a low echo, TPOAb, and tumor location were not significantly different between the two groups (P>0.05).

### Multivariate Logistic Regression Analysis

The medical information (predictors) with statistical significance in the univariate analysis was analyzed using a multivariate logistic regression analysis. The results confirmed that the following predictors were independently correlated with central CLNM (**Table 2**): positive TgAb (OR=1.964, p<0.05), microcalcifications (OR=2.839, p<0.001), and tumor size (>1.0 cm) (OR=2.814, p<0.001).

**Table 2:** Multivariate logistic regression analysis of the relationship between predictors of PTC (significant by univariate analysis) and central CLNM.

Predictors	OR	95% CI	P
TgAb			0.038
negative	Reference	Reference	
positive	1.964	1.039-3.711	
Microcalcifications			<0.001
absent	Reference	Reference	
present	2.839	1.684-4.787	
Tumor size (cm)			<0.001
≤1.0	Reference	Reference	
>1.0	2.814	1.634-4.848	
Age			0.080
<=40	Reference	Reference	
>40	0.627	0.369-1.056	
Blood flow signal			0.245
poor	Reference	Reference	
rich	1.409	0.791-2.509	
BRAF^V600E^			0.763
wild	Reference	Reference	
mutation	0.867	0.343-2.191	
Capsule contact			0.082
absent	Reference	Reference	
Present	0.590	0.195-0.830	

### Development of an Individualized Prediction Nomogram

**Figure 4:**
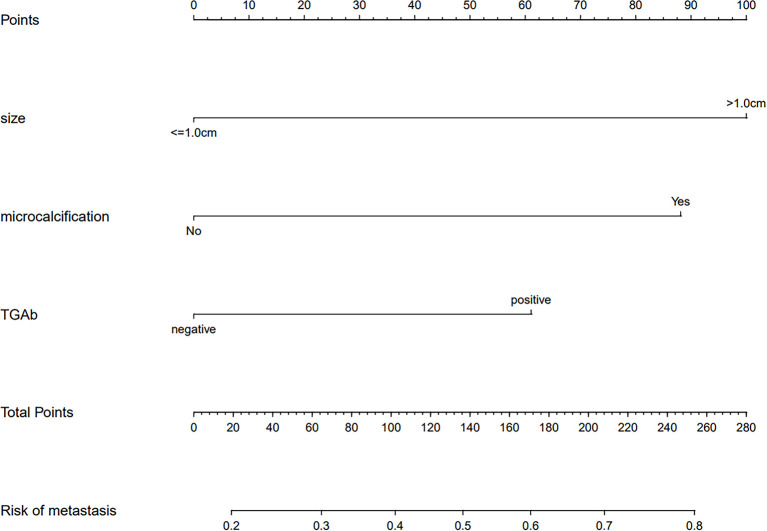
A prediction nomogram. The nomogram is used for the prediction of central CLNM in PTC patients (cN0). The prediction nomogram was developed in the cohort, with tumor size, US feature (microcalcifications), and thyroglobulin antibody (TgAb).

The results of the logistic regression analysis among tumor size (> 1.0 cm), US features (microcalcifications), and TgAb (positive) are given in **Table 2**. The model that involved the three independent predictors above was developed as a nomogram (**Figure 4**). In **Figure 4**, the tumor size (>1.0 cm) is assigned 100 points, whereas tumor size (≤1.0 cm) gets 0 points; a nodule with microcalcification is assigned 88 points, whereas a nodule without microcalcification gets 0 points; patients with positive TgAb is assigned 61 points, while those with negative TgAb score 0 points. The total points axis can reach up to a maximum of 249, and the prediction capability of metastasis risk ranges from about 0.20 to 0.80.

### The Validation of the Prediction Nomogram

**Figure 5:**
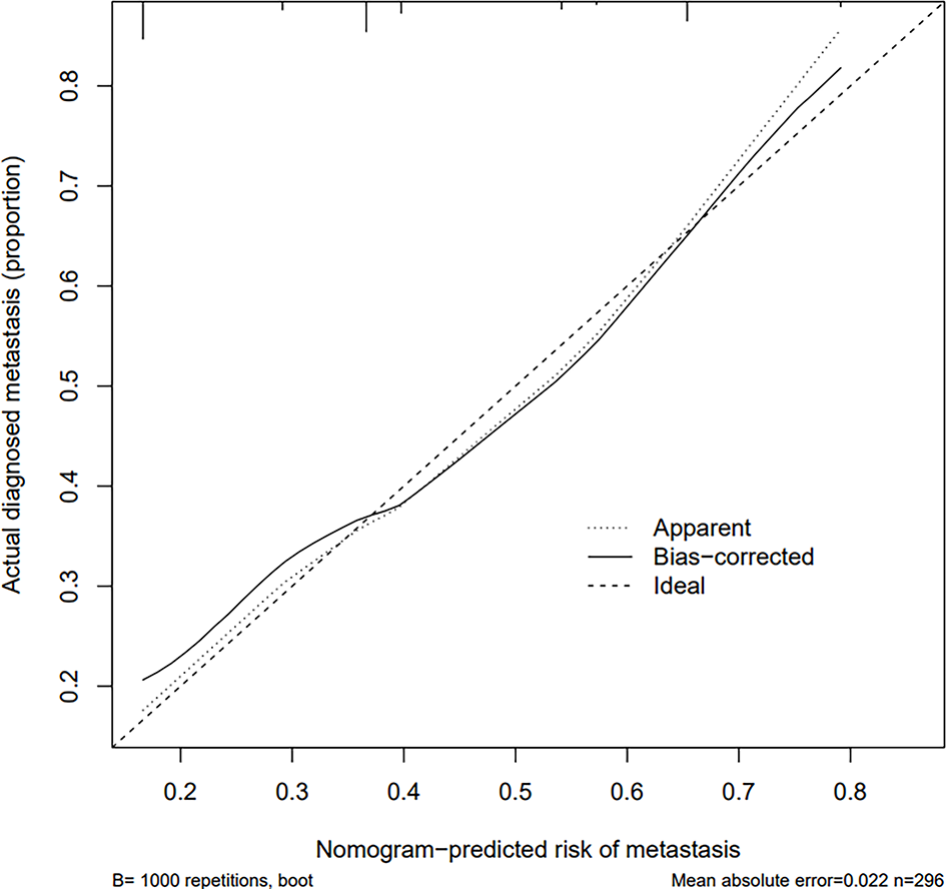
Calibration curves of the prediction nomogram. The solid line is close to the diagonal dotted line, which represents a good prediction ability. The x-axis represents the predicted central CLNM. The y-axis represents the actual central CLNM. The diagonal dotted line represents the perfect prediction by the ideal model. The solid line represents the performance of the nomogram, where a closer fit to the diagonal dotted line means a better prediction.

**Figure 6:**
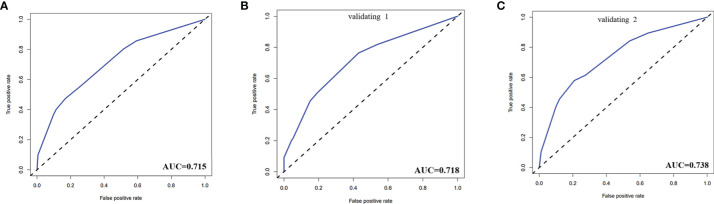
**(A)** The receiver operating characteristics (ROC) curve and area under the curve (AUC) in the training cohort. **(B)** ROC curve and AUC in the validating 1 cohort. **(C)** ROC curve and AUC in the validating 2 cohort. Validating 1 and validating 2 were performed to evaluate the accuracy of this model.

The calibration curve of the risk nomogram used to predict the risk of central CLNM in PTC patients (cN0) showed good consistency (**Figure 5**). The C-index for the prediction nomogram was 0.715 and further confirmed to be 0.718 and 0.738 through the two internal validations (Validation 1 and Validation 2, respectively) (**Figure 6**), which demonstrated the model**’**s good discrimination.

### Clinical Use

**Figure 7:**
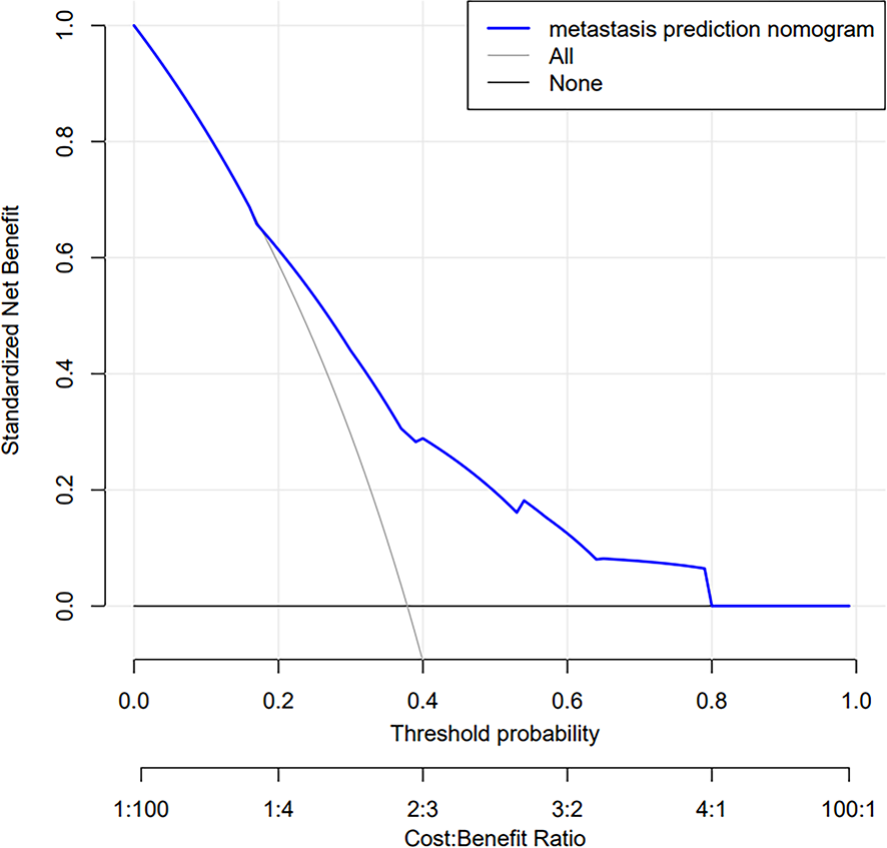
Decision curve analysis (DCA) for the risk nomogram. DCA shows that the model is clinically useful when intervention is decided in the threshold range of 16% to 80%.

The decision curve analysis (DCA) for the risk nomogram is presented in **Figure 7**. DCA showed that using this risk nomogram to predict central CLNM would be beneficial if the threshold probability was between 16% and 80%. In this range, according to the risk nomogram, the net benefit was comparable with several overlaps.

## Discussion

The incidence of thyroid carcinoma is increasing at an alarming rate, almost tripling every decade. In 2020, thyroid carcinoma was the fifth most common malignancy in women (25). Despite an excellent prognosis with a 10-year-survival rate exceeding 90% (26), previous studies have shown that 30% to 90% of PTC patients have lymph node metastasis, especially in the central region (27–29), which is one of the main causes of postoperative recurrence. A previous study suggested that the 3-year recurrence rate in the metastasis group was 4.54 times higher than that in the non-metastasis group, and although a good prognosis can still be achieved with reoperation, patients will be more likely to develop surgical complications (30, 31). Therefore, the management of the lymph nodes at the time of initial surgery is very important for the patient**’**s prognosis. Despite this, prophylactic RCLND is still controversial. Guo et al. suggest that prophylactic RCLND at the time of initial surgery may have benefits, including avoiding the high risk of permanent hypoparathyroidism and RLN injury associated with reoperation (31, 32). In contrast, Yoon et al. concluded that parathyroid hormone levels may be significantly reduced after CLND, and the risk of permanent hypocalcemia may increase (33). Therefore, PTC patients (cN0) benefit most from being able to predict central CLNM preoperatively accurately. Screening out reliable predictors from various medical information and establishing an effective prediction model are good solutions.

Our study included 11 clinical and US characteristics, four hematological indices (TSH, Tg, TgAb, and TPOAb), and BRAF^V600E^ status as potential predictors for central CLNM in PTC. Previous studies have reported sex, age, tumor size, tumor location, and microcalcifications as risk factors for CLMN (15, 18, 34, 35). In our study, age, blood flow signal, BRAF^V600E^ mutation, and capsule contact (the distance between the tumor and the capsule) were associated with central CLNM in PTC according to the univariate analysis. In contrast, they were not identified as independent factors through the multivariate logistic analysis. Finally, tumor size (> 1.0 cm), US features (microcalcifications), and TgAb (positive) were independent predictors for central CLNM according to the univariate and multivariate logistic regression analyses.

Previous studies have shown that thyroid microcalcifications are associated with central CLNM in PTC (15, 36, 37), and this was confirmed in this study. Microcalcifications are common in PTC and are considered to be a specific sign associated with PTC. They are mainly caused by small psammoma bodies 10-100 μm in diameter, which are usually round or concentric under light microscopy (38). s are deposits of calcium salts due to the proliferation of blood vessels and fibers, reflecting the rapid growth of cancer cells. Therefore, if microcalcifications were found in thyroid nodules by US, the central cervical lymph nodes should be assessed more carefully before and during the operation.

Tumor size has been considered an important predictor of central CLNM in PTC, but the thresholds are different. Chen et al. showed that tumor size ≤1 cm was a risk factor for central CLNM (37). However, more scholars believe that central CLNM is positively correlated with tumor size. Jiang et al. retrospectively studied 4107 PTC patients and found that tumor size (>0.5 cm) was an independent predictor of central CLNM (34). Moreover, Liu et al. concluded that a tumor size >1.0 cm was significantly associated with central CLNM in PTC (15). Our study also concluded that a tumor size >1.0 cm is the threshold for central CLNM. Nevertheless, the consensus is that the larger the tumor is, the greater the likelihood of metastasis to the central cervical lymph nodes.

Our study suggested that TgAb was also an independent predictor for central CLNM in PTC. A positive TgAb indicates that the tumor is active (39). Zhou et al. retrospectively studied 2926 papillary thyroid carcinoma (PTC) patients and found that Tg antibody positivity was an independent predictor of lymph node metastasis (LNM) (40). Vasileiadis et al. showed that the incidence of CLNM was significantly higher in PTC patients with positive TgAb (20.3%) than in patients with negative TgAb (10%) (41). Similarly, positive TgAb may be a risk factor for CLNM, and they observed a higher incidence of BRAF^V600E^ mutations in TgAb-positive patients than in TgAb-negative patients (42). BRAF is one of the three RAF genes of serine/threonine kinase (ARAF, BRAF, and CRAF), which is involved in signaling growth. BRAF is an important player in the mitogen-activated protein kinase (MAPK) pathway (43). The incidence of BRAF^V600E^ mutations has been reported to be 29%–69% in PTC (44). In our study, BRAF^V600E^ was included as a potential predictor of central CLNM, and the mutation incidence of PTC (CLNM group) was 85.29%, with a higher mutation incidence of 85.29% in the central CLNM group than in the non-CLNM group. However, it was not related to central CLNM according to the univariate and multivariate logistic analyses. Regarding its association with central CLNM, the findings to date have been inconsistent. Studies have shown that PTC combined with BRAF^V600E^ mutation is prone to lymph node metastasis, capsule invasion, and recurrence (37, 43, 45). In contrast, some studies suggest that the BRAF^V600E^ mutation is not associated with CLNM (46, 47).

Finally, we developed and validated a new prediction nomogram based on the three independent risk predictors described above, which facilitates the individualized prediction of central CLNM in PTC patients (cN0). Internal validation in the cohort showed good discrimination and calibration. DCA showed that using this nomogram to predict central CLNM would be beneficial if the threshold probabilities ranged from 16% to 80%. The development of this prediction model will enable individualized prediction of CLNM in most PTC patients (cN0), helping surgeons achieve accurate CLND for maximum patient benefit. The clinical application of the nomogram is convenient with simple addition calculation. For example, the PTC patient shown in **Figure 2**, had a 1.5 cm thyroid nodule with microcalcifications and positive Tg antibody. From **Figure 4**, it’s easy to get a total score of 249 points for the patient (size: 100 points, microcalcifications: 88 points, and positive Tg antibody: 61 points). Accordingly, we roughly estimated that the risk of central LNM was about 80%. The risk of central LNM was relatively high, and the RCLND was recommend, furtherly, the postoperative pathological result confirmed our prediction. Conversely, another PTC patient shown in **Figure 3**, had a 0.6 cm thyroid nodule with microcalcifications and negative Tg antibody. Her total score was calculated as 88 points, and it corresponded to approximately 36% of the central LNM risk. The risk is not high, and the decision of RCLND should be made more carefully. Conclusively, the postoperative pathological result confirmed non-central CLNM.

Our study also has some limitations. First, this was a retrospective study; selection bias is inevitable. Second, the number of PTC patients included in this study was small. In the future, we will accumulate more cases and perform more validation. Third, the US results are largely dependent on the diagnostic experience of the operator. Although experienced sonographers perform US, subjective factors may influence the data.

## Conclusion

In conclusion, this study screened the following three significant independent predictors for central CLNM in PTC patients (cN0): tumor size (> 1.0 cm), US features (microcalcifications), and TgAb (positive). We also developed a prediction nomogram based on the three independent predictors. The quantitative risk assessment and prediction provided by the nomogram can help surgeons assess the status of central cervical lymph nodes preoperatively accurately, avoid insufficient or excessive treatment (such as unnecessary prophylactic CLND), and provide a new strategy for the management of PTC patients (cN0).

## Data Availability Statement

The original contributions presented in the study are included in the article/supplementary material. Further inquiries can be directed to the corresponding author.

## Ethics Statement

The studies involving human participants were reviewed and approved by the Ethics Committee of the Second Affiliated Hospital of Chongqing Medical University. Written informed consent for participation was not required for this study in accordance with the national legislation and the institutional requirements.

## Author Contributions

LY and XG designed the study and wrote the main manuscript text. XG and WL provided the study materials or patients and analyzed all data. JC and LY revised the manuscript. All authors contributed to the article and approved the submitted version.

## Funding

This study was supported by the National Natural Science Foundation of China (grant no. 81701709), Chongqing Natural Science Foundation (grant no. cstc2020jcyj-msxmX2011), Science and Technology Bureau of Yuzhong District, Chongqing (grant no. 20200145), and Kuanren Talents Program of the Second Affiliated Hospital of Chongqing Medical University.

## Conflict of Interest

The authors declare that the research was conducted in the absence of any commercial or financial relationships that could be construed as a potential conflict of interest.

## Publisher’s Note

All claims expressed in this article are solely those of the authors and do not necessarily represent those of their affiliated organizations, or those of the publisher, the editors and the reviewers. Any product that may be evaluated in this article, or claim that may be made by its manufacturer, is not guaranteed or endorsed by the publisher.
